# AI-Augmented Prevention Science Needs Community-Engaged Prevention Science: a Framework for Greater Accountability

**DOI:** 10.1007/s11121-025-01842-7

**Published:** 2025-09-24

**Authors:** Eric Rice, Lindsay E. Young

**Affiliations:** 1 Center for AI in Society, University of Southern California, Los Angeles, CA 90089 USA; 2 Suzanne Dworak-Peck School of Social Work, University of Southern California, Los Angeles, CA 90089 USA; 3 Annenberg School for Communication and Journalism, University of Southern California, Los Angeles, CA 90089 USA

**Keywords:** Community-engaged research, Artificial intelligence, AI research, Prevention science, Research framework

## Abstract

With the ability to approximate human decision-making and to parse through large and complex data, artificial intelligence (AI) technologies such as machine learning algorithms stand to play important roles in diagnosing, treating, and preventing society’s most pressing social, health, and academic challenges. However, some AI applications for these ends have resulted in biased and discriminatory outcomes, demonstrating poor accountability to the communities these technologies impact and serve. The foundational premise of this paper is that AI-augmented prevention science needs community engagement to mitigate these harms. Drawing on a decade of work conducted at the University of Southern California’s Center for AI in Society, we present a model of community-engaged AI-augmented prevention science. What sets our formalization apart from previous frameworks for community-engaged AI research is its prevention science orientation. We highlight potential roles for community and AI at each stage of the prevention science life cycle, from problem conceptualization to intervention implementation, and delineate when community input can compel moments of critical retreat in that life cycle to remain accountable to community needs and values. We then illustrate how we integrated AI and community-engaged methods in our work to correct racial biases in a predictive risk algorithm used to prioritize vulnerable people experiencing homelessness for housing interventions. Our case study highlights two moments of critical retreat—informed by the values, experiences, and contextual knowledge of our community partners—that led to more equitable and inclusive outcomes. We conclude with recommendations for how to advance community-engaged approaches in AI prevention science research.

## Introduction

In the USA, we are facing a crisis of health and well-being. Still recovering from a global pandemic, we are also under siege by a cacophony of other chronic (e.g., heart disease, diabetes), infectious (e.g., hepatitis, HIV), and behavioral (e.g., depression, addiction) health challenges that are mounting extreme pressure on our healthcare system and cutting lives short. Although these health crises are preventable, their diagnosis and treatment are complex, as these health risks are often entangled with one another and layered into deeper social inequities (e.g., poverty) and systems of power and oppression (e.g., structural racism). Thus, to achieve the ambitious vision laid forth in the Healthy People 2030 initiative—to ensure “a society in which all people can achieve their full potential for health and well-being across the lifespan” (Huang et al., 2024, p. 1)—it is incumbent upon us to invest in prevention science methods that can parse through this analytic complexity to identify specific and actionable insights for person-centered care and generate solutions that are accessible to and inclusive of communities most impacted.

Artificial intelligence (AI)—i.e., the application of algorithms to approximate human cognition in analyzing complex health data and improving medical diagnosis, treatment, and service delivery (World Health Organization, 2024)—holds great promise for meeting prevention science’s analytic challenges. With the computational power of AI tools, data accessed from multiple sources, such as behavioral health surveys, smartphones and social media, and administrative data from clinical, criminal justice, and education settings, can be merged and integrated for tremendous risk assessment and decision-making potential (August & Gewirtz, 2019). For example, machine learning (i.e., the use of computers to learn from data and to identify patterns and make decisions based on those data without explicit instructions) has been used to identify risk and protective factors and to make predictions of risk associated with a spectrum of health outcomes and behaviors across chronic (Delpino et al., 2022), infectious (Chiu et al., 2022), and behavioral health (Shatte et al., 2019) domains. Natural language processing techniques (i.e., machine learning technologies that give computers the ability to interpret, manipulate, and comprehend human language) have been employed with large-scale social media data to detect early warning signs of disease outbreaks (Gupta & Katarya, 2020), to characterize public perceptions of health risks (Eysenbach, 2011), and to identify health misinformation (Schlicht et al., 2024). And on the intervention and implementation side, AI tools like machine learning decision aids, chatbots, and virtual coaches have been used to aid provider diagnoses (Jain et al., 2023) and to provide person-centered health support (Ding et al., 2023).

Prevention science studies the causes and prevention of social, health, and academic problems to improve the well-being of individuals, families, and communities (Society of Prevention Research, n.d). These problems disproportionately affect communities of color, economically disadvantaged groups, and other historically marginalized populations. For AI to address prevention science challenges, it must both benefit and be developed with these communities. We present a model for integrating community input into prevention research involving AI and related technologies. Using the Coordinated Entry System Triage Tool Research and Refinement (CESTTR) project as our case study, we show how our team of prevention and computer scientists partnered with community stakeholders to correct racial biases in a predictive risk algorithm used to prioritize people experiencing homelessness for housing services in Los Angeles.

### Community-Engaged Research

Toward meeting the access and inclusivity challenges of prevention science, studies show that engaging community members in the research process strengthens the development of interventions and the likelihood that they will be adopted and sustained (Israel et al., 1998). Community-engaged research—the process of working collaboratively with and through groups of people affiliated by place, special interest, or lived experience to address issues affecting the well-being of those people (Centers for Disease Control and Prevention, 1997)—is particularly important when working with culturally diverse and marginalized communities that, historically, have been failed by conventional, often paternalistic, approaches to health prevention (Graham et al., 2015). Community-engaged research prioritizes mutual benefit, co-learning, and shared decision-making throughout all stages of research, including defining research questions, selecting methods, collecting and interpreting data, and disseminating results. Moreover, community stakeholders can be involved in various roles, ranging from consultative (e.g., community advisory boards) to deeply collaborative (e.g., co-investigators or community-led initiatives) (Cargo & Mercer, 2008). By fostering the active engagement and influence of community stakeholders (e.g., community-based organizations, local government agencies, people with lived experience) in all phases of prevention research, an opportunity arises to engage local and contextual knowledge toward the development of a more relevant and useful prevention program, while also shrinking the trust gap between researchers and communities, and strengthening community capacity to sustain longer-term change (Israel et al., 1998).

### Biases in AI

Although AI methods offer tremendous promise, the perils of leveraging AI to solve societal challenges in the absence of community-based human safeguards are rapidly becoming known. Studies have shown that AI tools like risk assessment algorithms (i.e., machine learning or statistical models that estimate the likelihood of future outcomes), facial recognition algorithms (i.e., AI-driven systems that process digital images or video frames to detect, analyze, and match human faces against known face templates or databases), clinical decision support systems (i.e., computer-based systems that analyze patient-specific data to generate actionable guidance, alerts, or recommendations to support clinical judgment), and large language models (i.e., computer programs trained on large amounts of text so they can understand and generate human-like language responses) can reinforce or amplify inequities, particularly when the ground truth data they are trained on reflect those inequities (Rajkomar et al., 2018). In the case study we present, we discuss our work on a risk assessment algorithm to prioritize housing resources for people experiencing homelessness, and how we drew on community input in the development and implementation of this algorithm to ensure that its assessments were ethical and equitable.

Bias in AI can be defined as systematic errors in algorithmic decision-making processes that result in unfair outcomes (Ferrara, 2023). AI bias can take many forms, each with distinct sources and consequences. Historical bias stems from entrenched social inequalities embedded in the data that AI algorithms are trained on, for example, Amazon’s AI hiring tool, which downgraded resumes containing references to women because it had learned from male-dominated historical hiring patterns (Dastin, 2018). Representation bias occurs when certain groups are underrepresented in data, as seen in facial recognition systems from IBM and Microsoft that misclassified dark-skinned women at much higher rates due to inadequate racial and gender diversity in training images (Buolamwini & Gebru, 2018). Measurement bias results from flawed proxies, such as when the Correctional Offender Management Profiling for Alternative Sanctions (COMPAS) recidivism algorithm used arrest data—a biased measure influenced by systemic racism—to predict future crime risk, disproportionately labeling Black defendants as high risk (Angwin et al., 2016). Aggregation bias arises when models assume one-size-fits-all relationships, ignoring population heterogeneity. This is what occurred when historical health spending data were used to identify patients who would most benefit from a preventive health program, which systematically disadvantaged Black patients, as they spent less money on health care than comparably ill White patients (Obermeyer et al., 2019). Finally, deployment bias occurs when models are applied in real-world contexts that differ from their design assumptions, for example, when predictive policing tools like PredPol, trained on biased crime data, directed police to over-surveil communities of color, perpetuating cycles of injustice (Eubanks, 2018).

### AI Safeguards

Biases like these reveal what can happen when community stakeholders that stand to use or be affected by an AI application are left out of its conceptualization, design, and implementation. Conversely, a more responsible approach to AI research is one that anticipates and mitigates potential harms, prioritizes inclusive stakeholder engagement, and embeds social responsibility throughout the design process (Dignum, 2019). Thus, the foundational premise of this paper is that AI-augmented prevention science *needs* community-engaged prevention science to serve as a safeguard against AI bias and to ensure that AI solutions are designed with community needs and interests in mind.

As living safeguards, community members provide oversight, norms, and accountability, and contextual intelligence to AI systems. Safeguarding roles for community members tend to take three forms. First, diverse community stakeholders can serve on community advisory boards (CABs), which serve as bridges between AI researchers and the communities they aim to benefit. In so doing, CAB members help to translate community knowledge and values into research agendas and protocols. Second, community members can also be involved in human-in-the-loop validation, a process in which human judgment is integrated into the evaluation, refinement, or oversight of the AI system. For instance, community members can identify patterns of bias or harm that may be invisible to developers, or they can review model outputs in real-world scenarios to determine whether the AI system is making fair, accurate, and context-appropriate decisions. Finally, community stakeholders, including end-users, domain experts, and ethicists, can be part of a larger system of solicitation and feedback throughout the AI development process to help ensure that the system is aligned with real-world needs and ethical considerations.

Despite the need for these safeguards, community-engaged methods in AI research remain underutilized and underformalized. In their scoping review, Loftus et al. (2024) found that of approximately 10,880 articles describing AI applications in healthcare settings, only 21 (0.2%) described some form of community involvement. While all 21 studies made efforts to include data derived from community settings to optimize algorithmic performance, only one described the inclusion of community stakeholders in user-centered design, usability, or clinical implementation. No matter whether this reflects a lack of interest in community-engaged methods or a lack of training in how to employ them, the result is limited transparency regarding how to do this work.

### Purpose

In this paper, we aim to contribute to recent ad hoc efforts to articulate guiding principles for integrating community voices and values into AI research (e.g., Asabor et al., 2024; Bergman et al., 2024; Hsu et al., 2022; Lett & La Cava, 2023). However, what sets our model apart from those prior efforts is its prevention science orientation. In our model of community-engaged AI-augmented prevention science, we highlight potential roles for both AI and community at each stage of the prevention science “life cycle,” from problem conceptualization to intervention implementation, and underscore where community input compels moments of critical retreat in that life cycle to remain accountable to community needs. Moreover, as we will demonstrate, it is in these moments of retreat that responsible innovation occurs.

Our conceptualization of this model evolved from a decade of research conducted at the University of Southern California’s Center for AI in Society. Guided by the philosophy that AI should benefit our most vulnerable communities and, therefore, should be developed in partnership with those communities, the Center for AI in Society brings together computer scientists, social work, and behavioral health researchers, and community stakeholders to advance the scholarship and application of AI for community well-being. This ethos has driven our work in a variety of domains, including HIV (e.g., see Rice et al., 2021), substance use (e.g., see Negriff et al., 2022), homelessness prevention (e.g., see DiGuiseppi et al., 2020), housing allocation (e.g. see Rice et al., 2023), and suicide prevention (e.g., see Fulginiti et al., 2022).

## A Model of Community-Engaged AI-Augmented Prevention Research

**Fig. 1:**
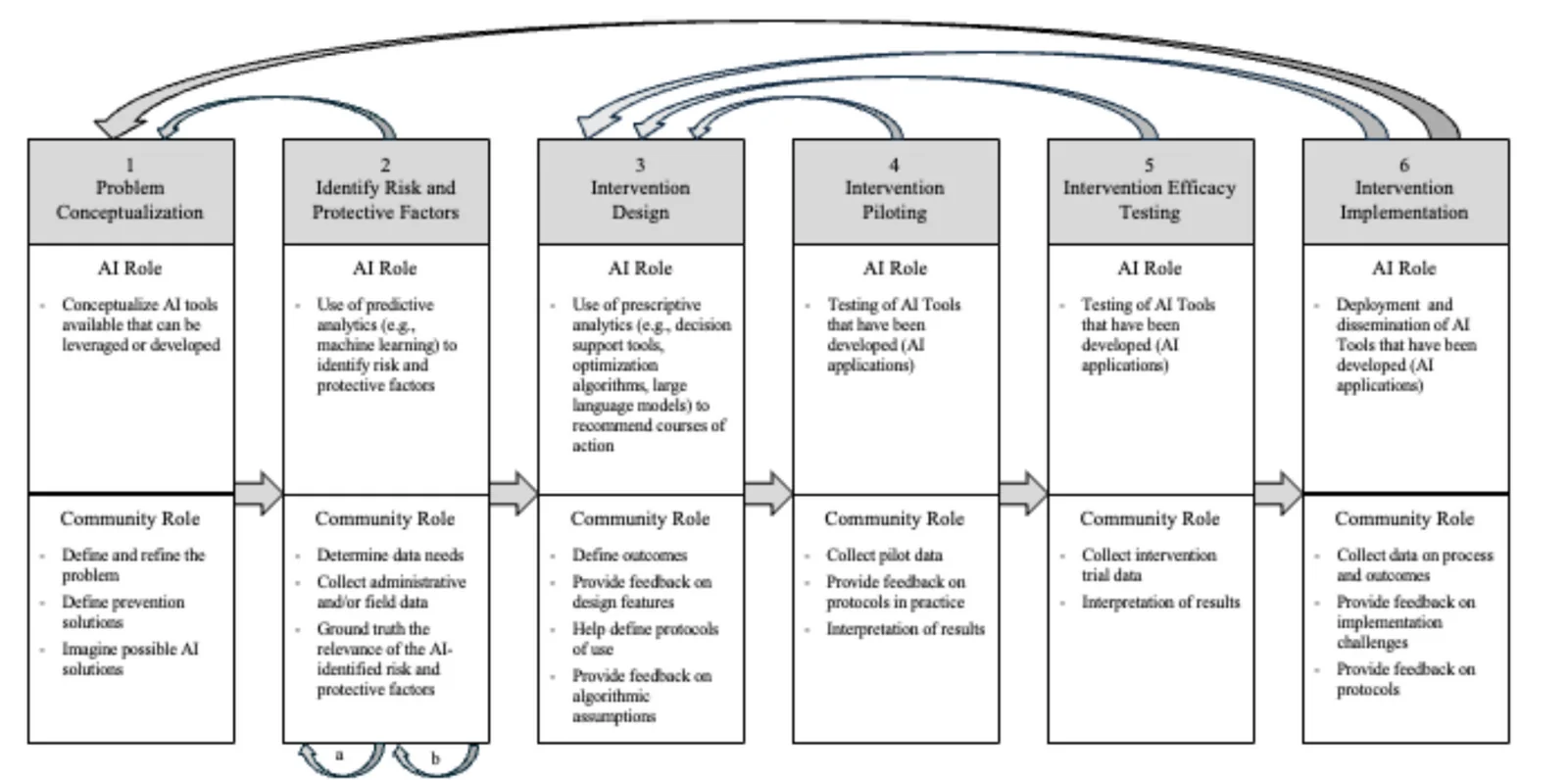
A sequential, yet iterative model of community-engaged AI prevention research that accounts for the possible roles of AI and community at each stage of the prevention science life cycle, from problem conceptualization to intervention implementation (columns 1–6). Curved arrows represent moments of critical retreat in which community input compels steps back in that life cycle or prolonged stays at a certain stage to remain accountable to community needs. Curved arrows labeled “a” and “b” (bottom) represent two moments of critical retreat that were critical to the case study discussed in this paper. Unlabeled curved arrows (top) represent moments of retreat that are likely to occur but were not present in the case study

The model proposed here spans what we call the prevention science life cycle, which is informed by Coie et al.’s (1993) Science of Prevention framework and the Preventive Intervention Research Cycle (Mrazek & Haggerty, 1994). Both frameworks posit that in prevention science, there is a concern for both observational research on risk and protective factors that impact prevention behaviors, and scientific development and testing of prevention interventions addressing specific behaviors for specific communities. In Fig. 1, we depict the relationship between the observational and intervention sides of prevention research as a sequential, yet iterative pathway advancing through six broad stages: 1) problem conceptualization, 2) identification of risk and protective factors, 3) intervention design, 4) intervention piloting, 5) intervention efficacy testing, and 6) intervention implementation. AI technologies, be they machine learning prediction models or large language models, can appear at every stage but need not (e.g., when the community, not AI, defines the problem at stage 1). Likewise, the community may not be engaged at every stage, although we argue that there is great value in involving the community in most stages.

There are three features of this diagram that we would like to underscore. First, AI-engaged research could happen at any of these stages. At the onset, a key conceptualization task when integrating AI into prevention science research is thinking through the AI tools and techniques that can be leveraged or developed to address the prevention science problem (stage 1). With large amounts of data to comb through, predictive analytics and machine learning often play key roles in identifying statistically meaningful risk and protective factors (stage 2) that can later be targeted in intervention work. The intervention itself can feature a wide variety of AI technologies, such as decision support tools, optimization algorithms, and large language models that can be used to recommend courses of action for clinicians and/or patients (stage 3), which then need to be tested and deployed in community settings (stages 4–6).

Second, AI-augmented prevention can and should engage the community at all stages of the prevention science life cycle. Community can help to conceptualize the prevention problem (stage 1) that needs to be solved (e.g., racial inequities in housing allocation). Toward identifying risk and protective factors, community stakeholders can collect and provide access to community-sourced data, help define and operationalize the risk and protective factors, and interpret their impact on key outcomes (stage 2). Moving to the intervention phases of the life cycle, community stakeholders can be involved in assisting the design of prevention interventions and providing feedback on necessary or desirable intervention modalities and components (stage 3). To meaningfully engage the target population and assist with the interpretation of study findings, the community is also a necessary partner when pilot testing and conducting efficacy trials (stages 4 and 5), and in the implementation of efficacious interventions at scale (stage 6). Moreover, like in stage 2, community stakeholders are often involved in the ongoing data collection that occurs during pilot, efficacy, and implementation stages of the intervention (stages 4–6).

All these statements are true whether one engages in AI-augmented work or simply community-engaged prevention science. In the context of AI-augmented work, however, we argue that community engagement is essential, as some communities may not trust AI or technology in general. In addition to trust, issues of data security, algorithmic bias/fairness, inclusion, and user preferences add to the complex set of design choices that impact the integration of AI and prevention science in community settings. As discussed above, we see communities as living safeguards who provide the needed oversight, norms, accountability, and contextual intelligence for AI systems. Community roles can take the form of CABs, human-in-the-loop validation, or a larger system of feedback and alignment. In our model, human-in-the-loop validation is most likely to occur during the identification of risk and protective factors (stage 2), intervention piloting (stage 4), and intervention implementation (stage 6), while CABs and systemic feedback systems have roles throughout the life cycle.

And third, while we present this life cycle as a sequential pathway, we acknowledge that community-engaged AI-augmented research often includes moments of critical retreat. Thus, the prevention life cycle is sequential, yet iterative, consisting of many possible feedback loops. In our experience, as the research unfolded and results were obtained, new problems and new solutions to those problems emerged. These moments of critical retreat often lead one to return to an earlier stage in the process and reevaluate previous decisions considering new information. This new information could come from study findings, logistic challenges, or emergent community insights and concerns.

In what follows, we draw on a case study—the Coordinated Entry System Triage Tool Research and Refinement (CESTTRR) study—to illustrate how our team of prevention scientists and computer scientists engaged with AI and community over the course of a three-year effort to revise and refine a risk assessment tool used to prioritize vulnerable individuals for housing resources in Los Angeles. Importantly, this case study, on its own, is not a full representation of the prevention science life cycle, as engagement with this process realistically involves multiple studies extended over longer periods of time. Instead, our case study allows us to illustrate how community engagement factored into stages 1, 2, and 4 of the model and how AI factored into stage 2 of the model. We also illustrate two moments of critical retreat (represented by arrows labeled “a” and “b”) that manifested in this work. Because critical moments of retreat are possible at almost every stage of the model, we include additional unlabeled arrows that represent moments of retreat that seem likely to occur but did not occur in the case study we discuss.

### Case Study

The Coordinated Entry System Triage Tool Research and Refinement study (March 2020 to May 2023) aimed to improve the use of triage tools used in Los Angeles’ Coordinated Entry System (i.e., the system that connects people experiencing a housing crisis to resources and housing). These triage tools are important, as they are used to prioritize the most vulnerable persons for limited housing resources and to prevent long-term adverse outcomes associated with homelessness (Rice et al., 2023). In our work, we used AI approaches to revise and refine an established triage tool used to identify risk factors for “adverse outcomes” (i.e., incarceration, hospitalization, psychiatric holds, death) among people experiencing homelessness in Los Angeles. With the understanding that structural racism impacts housing and homelessness in Los Angeles, particularly for Black people, our revisions to this tool were oriented around the need to reduce racial biases in those assessments and improve access to housing resources for Black, Latinx, and other racial/ethnic minority groups.

To ensure active community engagement throughout the research process, we first established a Community Advisory Board (hereafter CAB) composed of twenty-one persons with lived expertise of homelessness (73%), providers of homelessness services (59%), and employees of public agencies (32%) (these are not mutually exclusive categories). CAB members were compensated $100 for each two-hour meeting they attended, which included nine quarterly meetings with the research team and seven ad hoc working group meetings. Quarterly CAB meetings involved project updates and presentations of findings from the research team, and input from community members about outcome measures, algorithmic inputs, and data interpretation. Working group meetings were composed of CAB members and research staff who volunteered to work together on specific aspects of the project, for example, revising the phrasing of questions on the risk assessment questionnaire to reduce client stigma.

Second, we created a Core Planning Group made up of about 15 persons, including leadership from the Los Angeles Homeless Services Authority, the Los Angeles County Department of Mental Health, and the United Way Home for Good, as well as foundation funders, members of the Ad Hoc Committee on Black People Experiencing Homelessness, and members of the University of Southern California’s Homelessness Policy Research Institute. As described in our final report, both the CAB and Core Planning Group met continuously over a three-year period with approximately 24 convenings across these two groups (Rice et al., 2023). Amid the 24 convenings, approximately 12 of those sessions involved presentations by the research team to community partners to solicit specific feedback about key phases of the project. Presentations at the start and end of the project were also made to agency partner organizations, and several ad hoc presentations were made to specific stakeholder groups by request.

#### Problem Conceptualization

Los Angeles is home to one of the largest communities of persons experiencing homelessness in the country, with an estimated 69,144 people experiencing homelessness on any given day. Additionally, while 9% of the overall population is Black, Black people represent 30% of those experiencing homelessness (Los Angeles Homeless Services Authority, 2022). In response to this crisis, Los Angeles convened an ad hoc committee to address issues of structural racism in homelessness and housing. In December 2018, that committee released the “Report and Recommendations of the Ad Hoc Committee on Black People Experiencing Homelessness” (Los Angeles Homeless Services Authority, 2018), which called for: 1) research on the Vulnerability Index–Service Prioritization Decision Assistance Tool (hereafter VI-SPDAT; OrgCode Consulting, Inc., & Community Solutions, 2015) used to collect information about an individual’s vulnerabilities (e.g., history of homelessness, emergency service use, daily functioning challenges, mental health, and other behavioral risks) and the likelihood of needing specific housing interventions, and 2) how that tool may unequally impact Black people experiencing homelessness. To these ends, we were granted the opportunity to work with the Los Angeles Homeless Services Authority to investigate the accuracy of the VI-SPDAT tool, its fairness, and the processes by which it was administered with respect to issues impacting Black people.

#### Identify Risk and Protective Factors

The first task for the CAB and the Core Planning Group was to work with the research team to refine our work in creating a predictive risk algorithm using a traditional machine learning approach. In this approach, the algorithm first “learns” about patterns in existing data—a process known as the training phase. The resulting model is then applied to novel data to generate predictions about future outcomes or behaviors—a process known as the testing phase. The goal is to evaluate how well the model’s learned patterns generalize beyond the original data. We trained and evaluated an interpretable and well-established machine learning algorithm called the Least Absolute Shrinkage and Selection Operator (i.e., LASSO) regression. The data used for evaluating and improving the existing VI-SPDAT tool consisted of 71,747 assessments completed between July 2015 and October 2018, representing 54,543 unique persons, collected by the Los Angeles Homeless Services Authority in the Homeless Management Information System database. This database was linked to service utilization records from the Los Angeles County Chief Information Officer InfoHub, which contains data from eight county agencies, including the Department of Health Services, the Department of Mental Health, the Department of Public and Social Services, the Los Angeles Homeless Services Authority, the Sheriff’s and Probation Departments, and the Los Angeles County Department of Medical Examiner.

To work with such an algorithm, one must define an objective outcome, measurable with existing data, by which to assess the tool’s accuracy and bias. Presentations were made to both the Core Planning Group and CAB on alternative options, along with strengths and weaknesses related to measuring an outcome that captures homelessness “vulnerability.” The options under consideration relied on an integrated dataset so that the outcome measure could come from an agency other than the Los Angeles Homeless Services Authority. Based on that community process, the outcome variable was defined as “adverse outcomes,” a single binary indicator for the occurrence of one or more of the following events in a two-year window following assessment: (i) emergency or inpatient visits in Department of Health Services facilities; (ii) crisis stabilization episodes in Department of Mental Health facilities; (iii) justice involvement (Sheriff bookings or probation); (iv) substance use disorder diagnoses in the Department of Health Services or Department of Mental Health facilities; (v) death as recorded by the Medical Examiner-Coroner (Rice et al., 2023).

As one can already see, the nature of this community-engaged work extended beyond simple human-in-the-loop validation. Rather, our approach to community engagement centered community members as fundamental safeguards. Community drove the definition of the problem to be solved by AI (in the context of the original grant solicitation), community defined the outcome measure that was used in the LASSO regression (the specific AI modeling strategy), and, as we will show next, community provided feedback throughout the AI development process and, at times, compelled moments of critical retreat to ensure that the revised VI-SPDAT tool was aligned with the real-world needs of Black and Latinx people experiencing homelessness in Los Angeles.

#### A Moment of Critical Retreat

From the outset of this project, the community had defined the problem as being one of wanting an accurate and unbiased vulnerability tool. There was, however, a tertiary request, which was to reduce the burden for case managers and people with lived experience in the process of assessment, which was perceived as too lengthy. So, the initial model we presented to the community was the most parsimonious possible tool, with as few items from the original VI-SPDAT retained as possible, so as to minimize the time it would take to conduct the assessment. This initial tool included only nine items.

However, when we delivered this 9-item tool to the community, they expressed concern. From their perspective, there were fundamental flaws in its face validity; the nine items “felt” inadequate. From the perspective of our community stakeholders, several key issues, including substance use and mental health issues, were either entirely absent or missing key indicators. Without these indicators in the model, they were concerned that the risk assessment tool would miss vulnerabilities that many individuals experiencing homelessness are prone to struggle with. They thus initiated the first moment of critical retreat, as shown by arrow a in Fig. 1. This retreat was more like a prolonged stay in Stage 3 to accommodate the community’s desire to see these additional factors included in the model. This type of community engagement can be thought of as a human-in-the-loop validation process, one which requires community-based contextual understanding, which constitutes a critical community safeguard in the process of AI development.

In response to community feedback, we selected a larger number of potential questions by bootstrapping the modeling process (1000 iterations) and presenting to the community the full list of questions that had a nonzero coefficient in at least one bootstrap iteration. The purpose of using the bootstrap procedure was to identify policy-relevant variables that had been dropped from the initial modeling due to multicollinearity. The final revised tool consisted of a more inclusive list of 19 questions.

We then compared the accuracy and bias of the tool when using the 19-item assessment relative to the 9-item assessment. Accuracy is an assessment of how well the tool distinguishes between low- and high-risk participants, while bias reflects the degree to which members of certain groups (e.g., racial minorities) are incorrectly determined to be “not vulnerable” even when they are, a phenomenon called a false negative. For training and evaluating the predictive models, the data were randomly split into three partitions. Two of the three partitions were used during the model development and community collaborative phases in 2021–2022, using one partition to train the model and the other to test it. In this phase, metrics of prediction quality (e.g., accuracy, false negatives) were presented and discussed with community partners in interim presentations. The third data partition was held out in reserve to be used only for the generation of final evaluation metrics. That evaluation demonstrated that the 19-item assessment increased the tool’s accuracy by 11% and reduced race/ethnicity bias, as measured by the percentage of false negatives predicted by the model, from 5.9 to 0.7% for Black clients and 3.2 to 0.2% for Latinx clients (Rice et al., 2023). These results were shared with the CAB and the Core Planning Group in separate presentations, during which they each validated that the new 19-item tool with the improved performance metrics had an acceptable face validity and addressed their concerns regarding racial bias and fairness in the algorithm.

#### Another Moment of Critical Retreat

The community was now satisfied with the 19-item tool, but they were unhappy with how these data were being collected. Both the Core Planning Group and the CAB were concerned that the question wording and the face-to-face administration of the VI-SPDAT were creating undue barriers for clients. Specifically, they were concerned that clients from marginalized backgrounds, particularly Black clients, LGBQ clients, non-cisgender clients, and women, were distrustful of the assessment process and were under-reporting experiences of substance use, incarceration, and victimization. The procedures used in data collection, as well as the wording of specific items in the assessment, were both identified as problematic in this regard. Thus, a second moment of critical retreat was activated, as shown by arrow b in Fig. 1.

To tackle this, members of the research team and members of the CAB formed a working group to determine which of the remaining 19 questions required revision, based on several criteria, including concerns for equity, trauma experiences, sensitivity, and clarity. In working group sessions, CAB members rewrote the questions that were perceived to be problematic, after which the research team compiled several question variants and asked the CAB to vote for their preferred wording. We then tested the “winning” wordings in role-plays with CAB members. Subsequent revisions were made until the CAB and research team reached consensus on revised wording (Rice et al., 2023). Once again, our community partners ensured that the risk assessment process was aligned with the real-world needs of some of the most vulnerable individuals experiencing homelessness, and our CAB members helped to translate community knowledge and values into our research.

Simultaneously, we also conducted extensive one-on-one semi-structured interviews with service providers and persons with lived experience to discuss issues of tool administration. We then shared these results with the CAB and the Core Planning Group to further refine our understanding of tool administration issues (Rice et al., 2023). Based on their feedback, we then developed a list of priorities in tool administration refinements, which focused on reducing race- and gender-identity-based response biases, using trauma-informed practices, and building a sense of safety and trust. In particular, the CAB helped the team translate findings into practice, in some cases by making specific suggestions, and in other cases by role-playing and demonstrating to the team how such practices could be enacted. This led to specific areas of training and prompts built into the assessment tool to help case workers enact these revised practices.

#### Intervention Piloting

We then pre-piloted the newly reworded tool and its revised administration practices with the CAB. CAB members who were providers role-played with CAB members with lived expertise and demonstrated to the research team how to conduct appropriate interviews. The team worked with the CAB to refine these practices and developed a training for case managers based on this work. We then formally pilot-tested the new tool and new tool administration practices with nine community-based organizations across LA County. We found great acceptability with both providers and persons with lived experience (Rice et al., 2023).

This line of work has led to policy change in Los Angeles. At the time of writing of this paper, the Los Angeles Homeless Services Authority has moved to use the revised and refined assessment tool that we co-created with the community, including the more accurate, less biased scoring system that was a result of our AI work described above. The implementation of this tool and its subsequent impact on prioritizing individuals for housing resources remains to be seen; however, we are hopeful that Los Angeles will invest resources in a thorough evaluation of its implementation and make necessary adjustments that may arise to ensure that persons in need of housing resources are housed and that racial bias in this process is eliminated.

## Conclusion

In this paper, we argue for greater inclusion of community stakeholders in AI-augmented prevention research, such that community needs, values, and experiences guide its conceptualization, development, and implementation. In so doing, we position community engagement as a critical safeguard against algorithmic solutions that inadvertently reinforce the very same biases and patterns of systemic exclusion that often characterize the social, physical, mental health, and academic problems that prevention research aims to resolve.

To demonstrate how community-engaged AI prevention research can be done, we presented a conceptual model to articulate potential roles for both AI and community in each phase of the prevention science life cycle, and moments of critical retreat, where concerns of community stakeholders demand key revisions to ensure more inclusive and responsive AI solutions. We then illustrated how we partnered with community stakeholders to revise a risk assessment algorithm used to prioritize individuals experiencing homelessness for housing resources, with the goal of improving its ability to accurately capture the unique vulnerabilities experienced by Black people.

Critical to this project’s success was the fact that those closest to the prevention problem, namely Black people with lived experiences of homelessness and those on the front lines of providing homelessness services, held meaningful decision-making roles both on the CAB and in the Core Planning Group. This granted them the ability to push back on the research team and compel moments of pause or retreat when features of the revised tool were in question. For example, the community compelled a retreat when CAB members challenged the face validity of a more parsimonious version of the revised assessment tool that lacked appropriate indicators for key vulnerabilities like substance use and mental health issues. A second moment of retreat was activated when community stakeholders expressed concern that clients from marginalized backgrounds were distrustful of the assessment process and were under-reporting stigmatizing experiences. This led to a systematic effort to refine question wording and administration procedures with trust-building and trauma-informed principles in mind. Thus, each community-compelled retreat not only led to appreciable technical improvements (e.g., reducing algorithmic bias, improving client response rates) but also contributed to greater trust in the assessment tool itself, as the contextual knowledge and values of community members on the frontlines of homelessness had been operationalized into the algorithm and its implementation.

The lessons of our case study extend beyond the specifics of how to embed community perspectives in the development of a risk assessment algorithm for predicting homelessness vulnerabilities. With greater availability of large-scale administrative data across a variety of prevention science domains, including healthcare, education, and other social service settings, predictive risk models are playing more prominent roles in resource allocation and intervention decisions. Because many prevention science problems disproportionately impact marginalized populations, it is especially important to integrate community perspectives into the design of predictive risk models meant to address those disparities. Doing so not only ensures that these models are grounded in the lived experiences of those most affected, but also helps guard against reproducing inequities, misclassifications, and mistrust that can arise when communities are excluded from shaping tools that will directly influence their lives. Moreover, with its emphasis on continuous knowledge co-production between prevention science researchers and community stakeholders, our model of community-engaged AI-augmented prevention science provides a guide for making community-informed adjustments to ensure responsible AI that leads to more equitable, user-centered solutions in addressing health and social problems.

As public concerns mount regarding misguided and unethical applications of AI to societal problems (e.g., see Obermeyer et al., 2019; Angwin et al., 2016), we risk a breakdown in trust between communities and researchers and, therefore, losing opportunities to innovate for health prevention and equity. Therefore, we who see AI’s prevention science potential have an obligation to create a new narrative of AI-augmented research that reflects an ethos of responsibility, inclusion, and care for the communities we serve. To that end, it is incumbent that we do at least three things. First, within our own research teams, we must commit to a more intentional and self-conscious engagement with the community-engaged AI process, such that research is designed and implemented with the integration of both resources in mind. Using our conceptual model as a guide could help in this regard. Second, we must openly discuss and translate this work for wider audiences, such that lessons can be shared and public trust can be rebuilt. And third, we need to build transdisciplinary and community-embedded training environments, such that the next generation of computer scientists and prevention scientists receive cross-disciplinary pedagogical training and mentorship in a practical, problem-oriented research environment that teaches the importance and need for community involvement.
